# Mutagenic and physiological impacts of colchicine on growth, flowering behavior, and ISSR-based genetic variability in *Calendula officinalis* L

**DOI:** 10.1186/s12870-026-08536-4

**Published:** 2026-04-01

**Authors:** Makka A. Hassan, Hani S. Saudy, Mohammed Hewidy, Heba El-Sammak

**Affiliations:** 1 Department of Horticulture (Ornamental plants), Faculty of Agriculture Desert and Environmental, Matrouh University, Matrouh, Egypt; 2 Agronomy Department, Faculty of Agriculture, Ain Shams University, 68-Hadayek Shoubra, Cairo, 11241 Egypt; 3 Horticulture Department, Faculty of Agriculture, Ain Shams University, 68-Hadayek Shoubra, Cairo, 11241 Egypt; 4 Floriculture, Ornamental Horticulture and Landscape Gardening Department, Faculty of Agriculture, Alexanderia University, Alexanderia, Egypt

**Keywords:** Colchicine-induced deviations, coumarins, flavonoids, marigold flowering, terpenoids

## Abstract

**Purpose:**

Stimulating flowering ability is the main purpose of commercial development of ornamental plants. Colchicine could contribute to improve flowering and related physiological processes; however its potential mode on marigold (*Calendula officinalis*) as a marketable ornamental plant is unclear.

**Methods:**

The present study was carried out during two successive generations (M1 and M2) along two experimental seasons of 2019-20 and 2020-21 at the nursery conditions. Shoot-tip seedlings of marigold were treated by different concentrations of colchicine (0.00, 0.025, 0.050, 0.075 and 0.100%) to study its effect on the morphological traits, phytochemical composition as well as identifying the possibility of mutations induction using ISSR marker technique.

**Results:**

Non-significant variations in survival rates were obtained among the tested colchicine concentrations, while a noticeable decline in germination percentage was observed as colchicine concentration increased. Application of colchicine at concentrations of 0.025% and 0.075% possessed the highest values of total carbohydrates content and phenols content, respectively, in both M1 and M2 generations. In M1 generation, application of 0.025% colchicine was the superior treatment for stimulating all inflorescence attributes with lowering flowering period, while the colchicine concentrations of 0.075% or 0.100% showed the longest flowering period. In M2 generation, application of 0.100% colchicine achieved the highest values of flowering date and inflorescence attributes, while the longest flowering period was obtained with application of 0.025% colchicine. Both 0.050% and 0.100% concentrations showed abnormal and multiplied branching.

**Conclusion:**

Some variations in the habit of growth, leaf form and inflorescence structure were observed in both generations with application of colchicine. Commercially, using colchicine at 0.025% as growth stimulator is regarded as an effective practice for avoiding growth abnormality, while obtaining marketable marigold flowers. Also, ISSR marker- PCR technique was able to detect colchicine-caused mutations in marigold plant.

**Supplementary Information:**

The online version contains supplementary material available at 10.1186/s12870-026-08536-4.

## Introduction

Recently, there has been an increased interest in developing ornamental plant production systems [1–4]. This development has emerged through attempts to stimulate growth, productivity, and flower quality using various growth stimulants [5–10].

Marigold (*Calendula officinalis*) as a member of family ‘Asteraceae’ is an annual herb with yellow to orange flowers, originating from the Mediterranean region, and commonly known as pot marigold [11]. It grows in sun or partial shade and is easy to grow requiring little cultivation attention [12]. It is popular because it provides a lush color, aromatic scent and is used in the pharmaceutical, food, medicine industry and decoration of home gardens [11, 13]. Marigold contains volatile oils, carotenoids, carotene, lycopene and calendulin. It also contains saponins, resins, tannins mucilages, sterols, bitter principles, oleanolic acid, glucoronic acid, salicylic acid, violaxanthine, flovoxanthine, flavonoids, terpenoids and coumarins. Fresh blossoms contain azulenogenic sesquiterpene or sesquiterpene alcohol. Marigold has various uses; leaves are diaphoretic, diuretic, oxytocic, emmenagogue, astringent sedative, antiemetic, aromatic and antianemic. They are used as herbicide, assist healing of ulcers and astringent like hamamelis leaves. Flowers are used in case of dysmenorrhea and for the production of calendulin which is used in coloring food products as jellies and jams. The ray flowers are used to adulate saffron flowers which are very expensive [14, 15]. Extraction of glycosides from leaves and the petals has advantage in cycle of blood in the muscles. Glycosides are an important source for the national income of many countries in USA, Europe and Egypt [16].

The study of genetic diversity of species is important for plant breeding programs. In recent years, some studies have been conducted on the genetic diversity of *C. officinalis*. One PCR-based marker approach that contains multi-allelic loci, ISSR, can swiftly and informatively display the differences between closely related individuals. However, limited information is available on the simultaneous evaluation of colchicine-induced morphological, biochemical and ISSR-based genetic variation in marigold. Baciu et al. [17] studied the genetic diversity of *C. officinalis* by evaluating 34 genotypes of the species using the Random Amplified Polymorphic DNA (RAPD) technique. El-Nashar and Ammar [18] investigated the effects of colchicine on the molecular diversity of the species using sequence-related amplified polymorphism (SRAP) markers. The percentage of polymorphism ranged from 79% to 100% and the polymorphic information content (PIC) ranged from 0.85 to 0.97, demonstrating the effectiveness of SRAP markers in detecting the molecular diversity caused by colchicine treatment.

Mutation breeding is known tool for enhancing the genetic diversity in plants, and some mutations could induce new characters [19, 20]. Colchicine is an extremely poisonous alkaloid, originally extracted from *Colchicum autumnale* plants and has a mutagenic effect [21, 22]. The mutagenic effects generally result in shorter stems and larger inflorescences, fruits, and pollen grains [22]. Beside the phenotypic characters, the mutagenic effects can be evaluated more precisely by use of molecular marker techniques, where the molecular markers were used to detect genetic diversity among plant species [23]. Roychowdhury and Tah [24] reported that increasing the colchicine concentration from 0.1% to 0.7% for 10-hour treatment enhanced seed germination percentages. Although these concentrations were effective in improving germination behavior, the resulting seedlings were unable to survive. Pande and Khetmalas [25] found on stevia that germination percentages was decreased with increasing concentrations of colchicine mutagen, when seeds were subjected to varying concentrations from 0.250 to 0.5% for 12 and 24 h. Ramdas et al. [26] found on *Zanthoxylum armatum* that low germination percentage of soaked seeds in distilled water (0–15%) was achieved amongst the seedling exposed to 1.0 M colchicine treatment for 10–15 min. Xing et al. [27] soaked seeds of *Catharanthus roseus* in colchicine solution from 0.05 to 0.4% for 12 to 48 h. They found that the optimal treatment of induce tetraploid plants was 0.2% colchicine for 24 h and those plants had more branches and leaves. Maamoun [28] found on soaked dry seeds of *Nigella sativa* in aqueous 500 and 1000 ppm colchicine that significantly increase corrected germination rate index (CGRI) when compared with control, significantly decreased plant height, number of branches, number of flowers and flowering date compared to the control treatment in the M1-generation. Whereas 1000 ppm gave the tallest plant and the earliest flowering date, 500 ppm decreased the number of branches, number of flowers in the M2-generation. El-Nashar and Ammar [28] soaked seeds of *C. officinalis* in six different concentrations of colchicine (0, 400, 800, 1200, 1600, and 2000 ppm), for (1, 2, 3 and 4 h). Soaking seed in colchicine significantly enhanced both the fresh and the dry shoot and flowering date, number of flowers per plant, including flower diameter. The treatment of colchicine at 1200-ppm for 4-h, gave an effect on seed germination, whereas 800 ppm for 4 h produced the highest number of flowers and the largest flower diameter. The earliest flowering time was found at 800 ppm combined with a short soaking time (1 h), while the 4-h soaking time with 800 ppm, was recommended for growing *Calendula outdoors*, since it enhances flower development.

Different molecular marker techniques have been used to detect and evaluate genetic diversity such as amplified fragment length polymorphism (AFLP) markers [29], inter simple sequence repeat (ISSR) technique [30], random amplified polymorphic DNA (RAPD) technique [31] and ISSR technique [32]. However, limited information is available on the simultaneous evaluation of colchicine-induced morphological, biochemical and ISSR-based genetic variation in marigold.

Accordingly, the present work hypothesized that specific colchicine concentration could have a simulative influence on marigold physiology and related phenomena such as growth and flowering. Thus, the study was aimed to assess the potential genetic variation due to application of colchicine on the vegetative, flower yield components and phytochemical composition of marigold. The study was also extended to evaluate the potential genetic diversity owing to colchicine application using Inter-Simple Sequence Repeat (ISSR) technique.

## Materials and methods

### Experimental duration and location

The experiments in the present study were conducted over two successive generations (M1 and M2) along two experimental seasons of 2019/2020 and 2020/2021, in the Nursery of the Department of Floricultural, Ornamental Horticulture and Garden Design, Faculty of Agriculture, Alexandria University, Alexandria, Egypt. The study area is typical Mediterranean climatic conditions. The average air temperature, humidity, and solar radiation during the plant growth period (October-April) were approximately 25.0 °C, 52.4% and 28.7 MJ m^–2^ day^–1^, respectively.

### Trial layout and treatments application

A simple experimental layout was set in randomized complete block design with 3 statistical replicates; each one has 5 biological replications, involving ten plants of five colchicine treatments. Seeds of marigold were gained from Department of Ornamental Plants and Landscape Gardening Research, Horticulture Research Institute, ARC, Egypt. Seeds were sown on the 15th of September 2019 in 30-cm pottery pots containing a mixture of sand and peat moss (1:1 v/v). At 4–6 leaf old, 35 days after sowing (DAS), plantlets apical-meristem was treated with 5 ml aqueous colchicine per each plantlet for 3 times, weekly-intervals. Fresh solutions from colchicine were prepared at the concentrations of 0.025, 0.050, 0.075 and 0.100% and control (distilled water). Next, seedlings were individually transplanted into 30-cm diameter pots containing the soil mixture of clay and sand (1:1 v/v). For growing the M2 generation, seeds were collected from plants grown in the first season for each treatment and then sown on the 15th of September 2020.

### Assessments

Survival and germination percentages were calculated based on 10 plants per replicate in three replicated.

At the end of the experiment, survival percentage for M1 was measured according to formula 1.

1 $$\mathrm{S}\mathrm{u}\mathrm{r}\mathrm{v}\mathrm{i}\mathrm{v}\mathrm{a}\mathrm{l}\mathrm{\%}=\frac{\mathrm{N}\mathrm{u}\mathrm{m}\mathrm{b}\mathrm{e}\mathrm{r}\mathrm{o}\mathrm{f}\mathrm{s}\mathrm{u}\mathrm{r}\mathrm{v}\mathrm{i}\mathrm{v}\mathrm{e}\mathrm{d}\mathrm{p}\mathrm{l}\mathrm{a}\mathrm{n}\mathrm{t}\mathrm{s}}{\mathrm{N}\mathrm{u}\mathrm{m}\mathrm{b}\mathrm{e}\mathrm{r}\mathrm{o}\mathrm{f}\mathrm{t}\mathrm{r}\mathrm{a}\mathrm{n}\mathrm{s}\mathrm{p}\mathrm{l}\mathrm{a}\mathrm{n}\mathrm{t}\mathrm{e}\mathrm{d}\mathrm{s}\mathrm{e}\mathrm{e}\mathrm{d}\mathrm{l}\mathrm{i}\mathrm{n}\mathrm{g}\mathrm{s}}\times100$$

At 30 DAS, seed germination percentage for M2 according to the following formula 2.

2 $$\mathrm{S}\mathrm{e}\mathrm{e}\mathrm{d}\mathrm{g}\mathrm{e}\mathrm{r}\mathrm{m}\mathrm{i}\mathrm{n}\mathrm{a}\mathrm{t}\mathrm{i}\mathrm{o}\mathrm{n}\mathrm{\%}=\frac{\mathrm{N}\mathrm{u}\mathrm{m}\mathrm{b}\mathrm{e}\mathrm{r}\mathrm{o}\mathrm{f}\mathrm{g}\mathrm{e}\mathrm{r}\mathrm{m}\mathrm{i}\mathrm{n}\mathrm{a}\mathrm{t}\mathrm{e}\mathrm{d}\mathrm{s}\mathrm{e}\mathrm{e}\mathrm{d}\mathrm{s}}{\mathrm{N}\mathrm{u}\mathrm{m}\mathrm{b}\mathrm{e}\mathrm{r}\mathrm{o}\mathrm{f}\mathrm{t}\mathrm{o}\mathrm{t}\mathrm{a}\mathrm{l}\mathrm{s}\mathrm{e}\mathrm{e}\mathrm{d}\mathrm{s}}\times100$$….

### Physio-biochemical constituents in leaf

For estimating plant physiological (plant pigments) and biochemical constituents (total soluble carbohydrates and phenols), plant leaves samples were collected at 90 DAS when the plants were at vegetative stage.

### Plant pigments

Based on the formulas 3–5, described by Moran [33] chlorophylls and carotenoids were estimated. The leaf samples of 500 mg fresh weight were extracted with 10 ml of N, N-Dimethylformamide kept for 24 h in a dark place at 4 °C until all pigments were extracted. Then, the extracts were centrifuged for 10 min at 8000 rpm. The pigment quantification was performed by spectrophotometry, at wavelengths of 664, 647 and 480 nm for chlorophyll a, chlorophyll b and carotenoids, respectively.

…

3 $$\mathrm{C}\mathrm{h}\mathrm{l}\mathrm{o}\mathrm{r}\mathrm{o}\mathrm{p}\mathrm{h}\mathrm{y}\mathrm{l}\mathrm{l}\mathrm{a}\mathrm{c}\mathrm{o}\mathrm{n}\mathrm{c}\mathrm{e}\mathrm{n}\mathrm{t}\mathrm{r}\mathrm{a}\mathrm{t}\mathrm{i}\mathrm{o}\mathrm{n}\left(\mathrm{m}\mathrm{g}\mathrm{g}{\mathrm{F}\mathrm{W}}^{-\hspace{0.17em}1}\right)\hspace{0.17em}=\hspace{0.17em}12.64\mathrm{*}{\mathrm{A}}_{664}\--2.99\mathrm{*}{\mathrm{A}}_{647}$$

4 $$\mathrm{C}\mathrm{h}\mathrm{l}\mathrm{o}\mathrm{r}\mathrm{o}\mathrm{p}\mathrm{h}\mathrm{y}\mathrm{l}\mathrm{l}\mathrm{b}\mathrm{c}\mathrm{o}\mathrm{n}\mathrm{c}\mathrm{e}\mathrm{n}\mathrm{t}\mathrm{r}\mathrm{a}\mathrm{t}\mathrm{i}\mathrm{o}\mathrm{n}\left({\mathrm{m}\mathrm{g}\mathrm{g}\mathrm{F}\mathrm{W}}^{-\hspace{0.17em}1}\right)\hspace{0.17em}=\hspace{0.17em}23.26\mathrm{*}{\mathrm{A}}_{647}\--5.6\mathrm{*}{\mathrm{A}}_{664}$$

5 $$\mathrm{C}\mathrm{a}\mathrm{r}\mathrm{o}\mathrm{t}\mathrm{e}\mathrm{n}\mathrm{o}\mathrm{i}\mathrm{d}\mathrm{s}\mathrm{c}\mathrm{o}\mathrm{n}\mathrm{c}\mathrm{e}\mathrm{n}\mathrm{t}\mathrm{r}\mathrm{a}\mathrm{t}\mathrm{i}\mathrm{o}\mathrm{n}(\mathrm{m}\mathrm{g}\mathrm{g}\mathrm{F}\mathrm{W}-\hspace{0.17em}1)=\frac{1000*{\mathrm{A}}_{480}-0.89*Chla}{245}$$

### Total soluble carbohydrates

Total soluble carbohydrates content was determined according to Hedge and Hofreiter [34]. Estimation of Total carbohydrates; 1 g of air-dried samples was submerged overnight in 10 ml of 80% (v/v) ethanol at 25 °C with periodic shaking. The ethanolic mixture was filtered and the ethanolic filtrate was made up to known volume. Carbohydrates and first hydrolyzed into simple sugars using dilute hydrochloric acid. In hot acidic medium glucose is dehydrated to hydroxymethyl furfural. This compound forms with antherone a green-coloured product with an absorbtion maximum at 630 n.m. standard curve was prepared of glucose. Amounts of carbohydrate present in 100 g of the sample = mg of glucose / volume of test sample x 100.

### Total phenolic contents

Total phenolic content was determined using the Folin–Ciocalteu method as described by Singleton and Rossi [35]. The free and bound phenolic extracts were diluted to an appropriate concentration. The diluted solution 0.5 ml was then oxidized with folin–ciocalteu reagent (0.5 ml) and then neutralized with saturated 1 ml of 25% (w/v) sodium carbonate (Na_2_CO_3_) solution. The volume was adjusted to l0 ml with distilled water, then thoroughly mixed and allowed to stand for 45 min at ambient temperature. The solution was centrifuged for 5 min at 4000 g, and the absorbance of the clear supernatants was measured at 725 nm using a spectrophotometer (Unico^®^ 1200 spectrophotometer). A standard calibration was prepared using ferulic acid (0, 20,40,60,80 and 100 mg/ml) and the content of total phenolics in each extract was calculated and expressed as mg of ferulic acid equivalent (GAE)/g of the sample. Total phenolic contents in plant extracts was expressed in gallic acid equivalents (GAE) and was calculated using the following equation: C = cV/m (12), where C is the total content of phenolic compounds, mg GAE /g dry extract; c the concentration of gallic acid obtained from the calibration curve, mg/ ml, V the volume of extract ml and m is the weight of extract, g.

### Physio-biochemical constituents in inflorescence

Physio-biochemical constituents in inflorescence were determined as beta carotene content, total coumarins content, total terpenoids, and total flavonoid.

### Β-Carotene was estimated using

A standard stock solution was prepared by dissolving 1.5 mg of β-carotene in 250 ml of acetone. From this, a series of working standards were derived to establish a calibration curve at a wavelength of 450 nm. Plant samples (dry flower rays) were processed into a paste with acetone, filtered, and diluted to volume before spectrophotometric measurement to determine the concentration per gram of dry weight as described by AOAC [36].

### Total coumarins

Total coumarins content was determined using spectrophotometric method described by Wierzchowska and Stecka [37]. For the analysis, 0.8 g of raw material was extracted in 100 ml of chloroform for 4 h in Soxhlet apparatus. The obtained extract was measured at a wavelength of 314 nm. The results were calculated in mg/g DW.

### Total terpenoids

Total terpenoids was determined following methanol extraction, the addition of chloroform and concentrated sulfuric acid produced a characteristic reddish-brown color. The intensity of this reaction was measured at 538 nm to quantify total terpenoid content as described by Wadood et al. [38].

### Total flavonoid content

Total flavonoid content was extracted via cold maceration in methanol for three days. The flavonoid concentration was determined using the aluminum chloride colorimetric method. Depending on the reference standard used, absorbance was measured at either 430 nm (for Quercetin equivalents) or 510 nm (for Catechin equivalents) following the addition of specific reagents including sodium nitrite and sodium hydroxide as determined by Munhoz et al. [39].

### Vegetative and flowering traits

At maturity (120 DAS) for M1 and M2 generations growth and floral traits were recorded. Vegetative traits, i.e. plant height, stem diameter, branches numbers plant^− 1^, leaves number plant^− 1^, leaf area [40], and fresh and dry weights of vegetative growth were estimated. Regarding flowering attributes, the following parameters were recorded at the full opening stage: flowering date (days to first inflorescence emergence), inflorescences number plant^− 1^, the diameter and stalk length of the inflorescences. Additionally, the fresh and dry weights were determined using 10 inflorescences per treatment. Stalk measurements were taken from the disk flower to the first set of true leaves on the flowering stem. Further, flowering period was assessed (expressed in the number of days of flowering from the appearance of the first inflorescence on each plant until the appearance of the last one).

### Abnormal growth

All plants of the different treatments in both generations (M1 and M2) were examined to search for the abnormalities and changes in the vegetative and flowering growth). These changes included growth behavior, and the overlap of color between both leaves and inflorescences.

### Inter-simple sequence repeat (ISSR) analysis

ISSR analysis was done as described by Shaw et al. [41]. For ISSR analysis, five selected inter simple sequence repeats were used for PCR amplification. Each amplification reaction mixture of 25 µL contained 25 ng template DNA, 2.5 µL of 10X assay buffer (100mM Tris-HCl pH 8.3, 500 mM KCl and 0.1% gelatin), 1.5 mM MgCl2, 200 µM of each dNTPs, 15 ng primer and 0.5U Taq DNA polymerase. Total genomic DNA was amplified through GeneAmp PCR System cycler as follows; 2 min denaturation step at 94° C, followed by 40 cycles of denaturation at 94 ° C for 20 s, annealing at required temperature for 30 s, extension 72° C. The primers name, sequences and annealing temperature are illustrated in Table 1.

**Table 1:** Inter Simple Sequence Repeat (ISSR) primers features used to analyze genetic relationships among the control and colchicine-treated marigold plant samples

Primers	Primer sequence	Annealing temperature °C
IG-03	5’GAGGGTGGAGGATCT-3’	48
IG-09	3’-(AG)8 C-5’	52
IG-12	3’-(GA)8 C-5’	52
IG-13	3’- (GA) 8 A-5’	50
IG-11	3’- (AG) 8 C-5’	52

Source: [107]

The amplicons were separated in 1.5% agarose gel for ISSR. Electrophoresis was performed at a constant voltage at 80 Volts for 100 min. The amplicons were visualized under UV light and photographed. Gel documentation system (Geldoc-it, UVP, England), was applied for data analysis to estimate fragment sizes were used to assign loci for each primer; bands were scored as diallelic for each assigned locus (1 = present; 0 = absent). Similarity coefficient was measured then, phylogenetic tree of Nei’s genetic distances were constructed based on presence–absence data matrix was analyzed with Totallab analysis software, www.totallab.com, (Ver.1.0.1).

### Statistical analysis

Each data set at significance level of *p* ≤ 0.05 was subjected to the Shapiro-Wilk’s test before analysis of variance technique (ANOVA) to determine if the data exhibited a normal distribution. Moreover, the homoscedasticity assumption was verified by Levene test. Once these assumptions were statistically validated, the measured data were statistically analyzed based on the ANOVA using SAS program, version 9.47 (2020) statistical software. For post-ANOVA-mean separation, least significant difference test (LSD) (*p* ≤ 0.05) was used [42]. For the percentage data on seed germination and plant survival, an angular transformation was applied, and all statistical analyses were performed using the transformed values. Furthermore, DNA isolated and responsive genes were presented by showing hierarchical clustering diagram of transcriptional expression.

## Results

### Survival and germination percentages

**Fig. 1:**
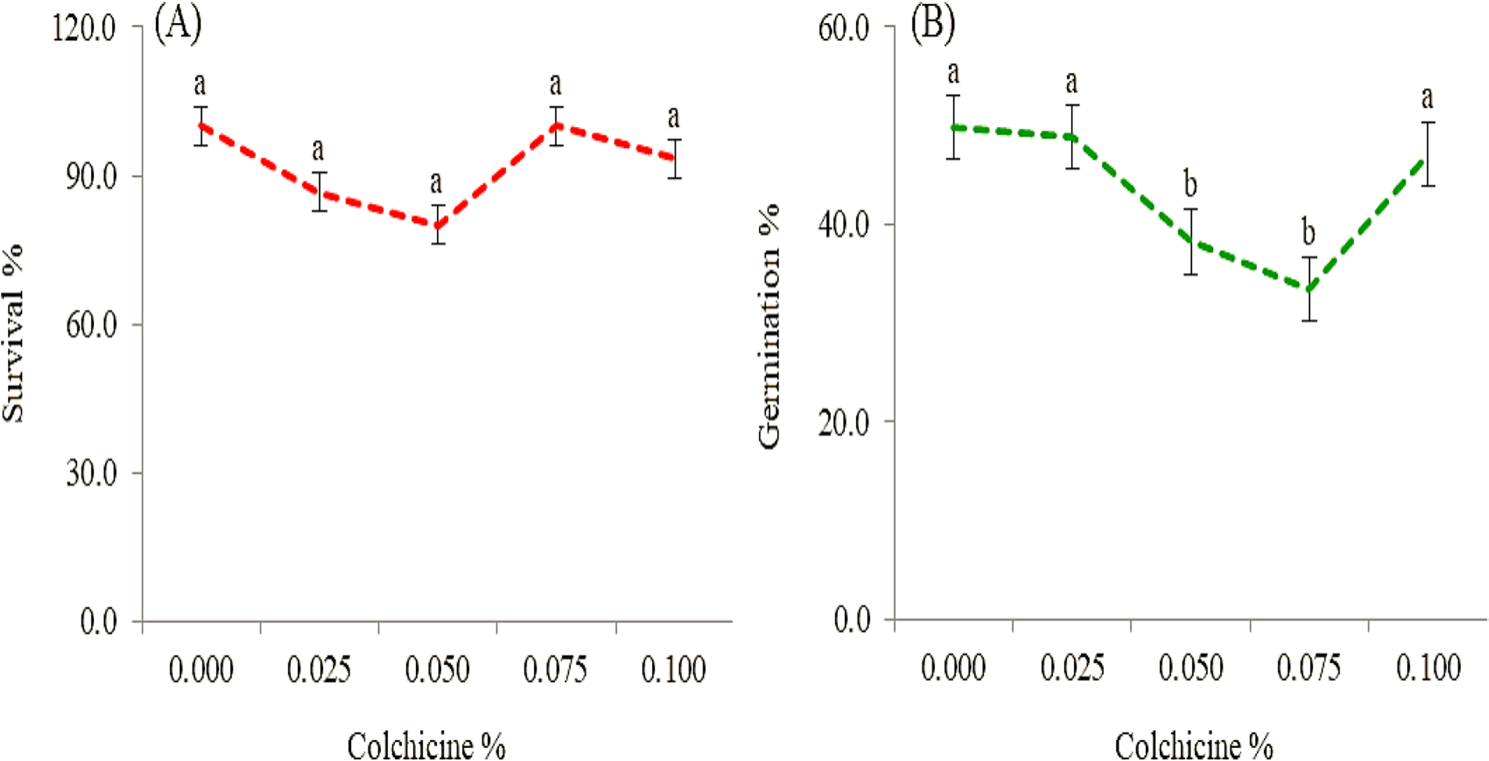
Survival % (**A**) at the first generation and seed germination % (**B**) at the second generation of marigold as affected by colchicine concentration. Values marked with the different alphabetical letters within group of means refer to significant variations, using least significant difference (LSD) test at the 0.01 level of probability

Survival percentage is a critical parameter in evaluating the efficiency of polyploid induction following the injection of colchicine solution into the shoot tips of plants. The obtained results indicated that there is no significant variation in survival rates among the tested concentrations (Fig. 1-A).

Seed germination tests were conducted using seeds obtained from the M1 generation of marigold which had been treated with different concentrations of colchicine. The control treatment (untreated seeds) recorded the highest germination percentage at 49.82%, significantly outperforming all colchicine-treated groups (Fig. 1-B). A noticeable decline in germination percentage was observed as colchicine concentration increased, indicating a dose-dependent inhibitory effect on seed viability and early seedling development.

Germination tests on marigold M1-seeds revealed a pronounced inhibitory effect of colchicine treatment (Fig. 1-B). Untreated controls exhibited maximal germination (49.82 ± 1.7%), significantly outperforming all colchicine-treated groups (*p* < 0.01). Germination rates declined progressively with increasing colchicine concentrations, demonstrating a clear dose-dependent response. Early seedling development was similarly impaired, with treated groups showing reduced radicle emergence and cotyledon expansion. This discrepancy may stem from differences in species-specific responses or experimental conditions such as colchicine concentration, treatment duration, and seed viability.

### Physio-biochemical constituents in leaf

#### Plant pigments

All plant pigments were substantially influenced by colchicine concentrations in M1 and M2 generations, except chlorophyll b and total carotenoids in M2 generation (Table 2). Herein, the highest colchicine concentration, i.e. 0.100% gave the maximal values of chlorophyll a in M1 and M2 and chlorophyll b and total carotenoids in M1. However, the difference between 0.100% and each of 0.025% or 0.075% (for chlorophyll a and total carotenoids in M1), 0.025% (for chlorophyll a in M2), and 0.00% or 0.075% (for chlorophyll b) were not significant.

**Table 2:** Mean values of plant pigments of marigold leaf as affected by colchicine concentration in the first (M1) and second (M2) generations

Colchicine %	Chlorophyll a (mg g^− 1^ FW)		Chlorophyll b (mg g^− 1^ FW)		Total carotenoids (mg g^− 1^ FW)	
	M1	M2	M1	M2	M1	M2
0.000	0.754^c^	0.576^b^	0.242^ab^	0.200 ^a^	0.178^b^	0.145 ^a^
0.025	0.904^ab^	0.596^ab^	0.151^bc^	0.212^a^	0.230^a^	0.147 ^a^
0.050	0.848^b^	0.585^b^	0.118^c^	0.180 ^a^	0.173^b^	0.142 ^a^
0.075	0.959^a^	0.554^b^	0.172^abc^	0.194 ^a^	0.208^ab^	0.138 ^a^
0.100	0.899^ab^	0.658^a^	0.264^a^	0.222 ^a^	0.202^ab^	0.161 ^a^
LSD_5_	0.091**	0.063*	0.093*	NS	0.043*	NS

Values marked with the different alphabetical letters within group of means refer to significant variations, using least significant difference (LSD) test; * and *= Significant at the 0.05 and 0.01 level of probability; *NS* Not significant

#### Total soluble carbohydrates and phenols

**Fig. 2:**
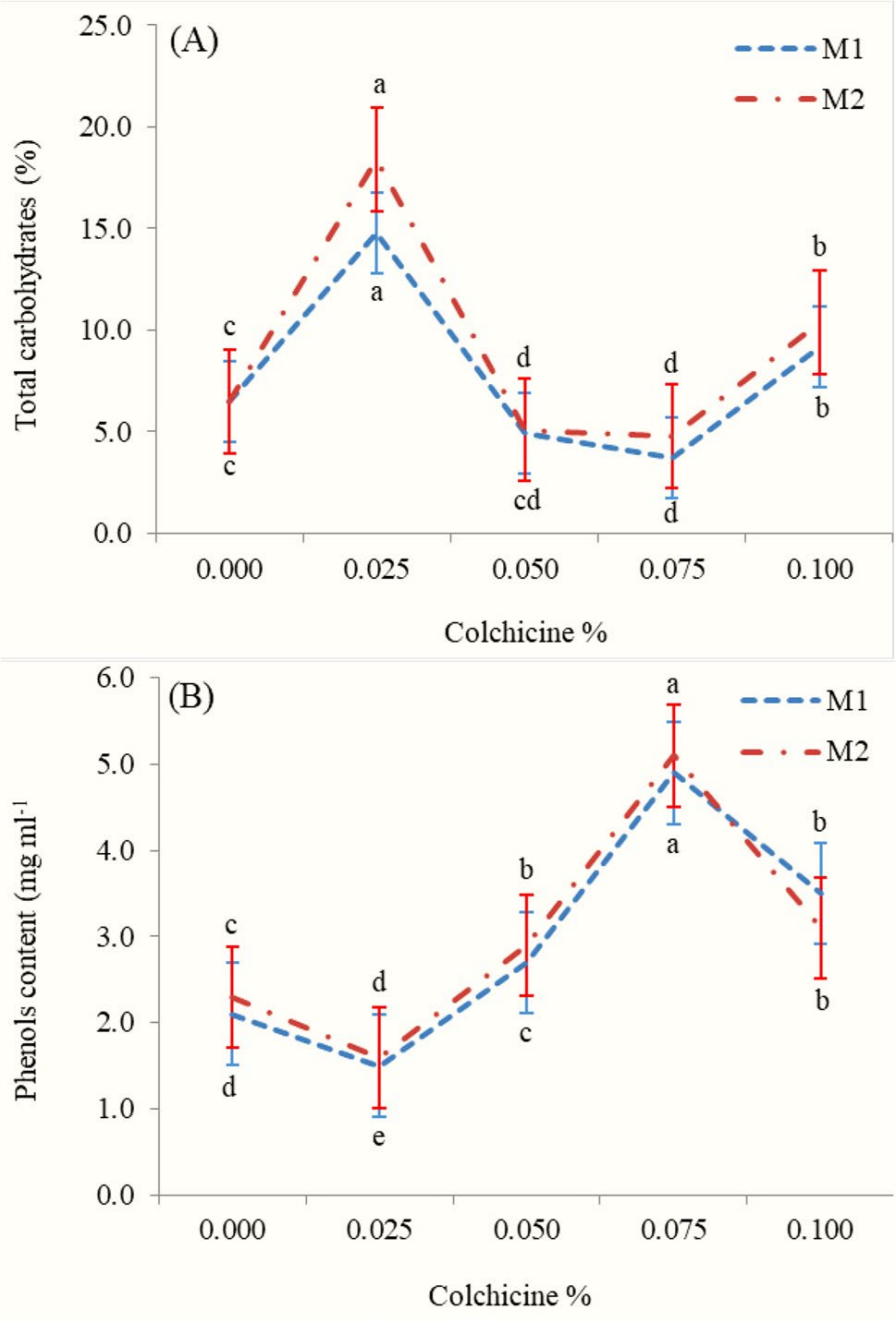
Mean values of total carbohydrates (**A**) and phenols content (**B**) of marigold leaf in the first (M1) and second (M2) generations as affected by colchicine concentration. Values marked with the different alphabetical letters within group of means refer to significant variations, using least significant difference (LSD) test; * and **= Significant at 0.01 level of probability

As for total carbohydrates and phenols contents, application of colchicine at concentration of 0.025% possessed the highest increases in total carbohydrates content outperforming the other concentrations in both M1 and M2 generations (Fig. 2-A). While the maximum value of phenols content was obtained with colchicine at concentration of 0.075% in both M1 and M2 generations (Fig. 2-B). On the contrary, applying concentration of 0.050% or 0.75 (for total carbohydrates content in both generations) as well as 0.00% in M1 and 0.025% in M1 (for phenols content) resulted in the lowest values.

### Biochemical constituents in inflorescence

As shown in Table 3, all assessed biochemical constituents of marigold inflorescence were markedly affected by colchicine concentration in the first (M1) and second (M2) generations. Beta carotene (with 0.025% colchicine), total coumarins and total flavonoids (with 0.025% or 0.050% colchicine) and total terpenoids (with 0.050% colchicine) resulted in an increase in M1 generation. While, the highest increase of beta carotene (with 0.100% colchicine), total coumarins (with 0.025%, 0.050% or 0.100% colchicine), total terpenoids (with 0.050% colchicine) and total flavonoids (with 0.025% or 0.050% colchicine) were obtained in M2 generation.

**Table 3:** Mean values of biochemical constituents of marigold inflorescence as affected by colchicine concentration in the first (M1) and second (M2) generations

Colchicine %	Beta carotene (mg g^− 1^ DW)		Total coumarins (mg g^− 1^ DW)		Total terpenoids (mg g^− 1^ DW)		Total flavonoids (mg g^− 1^ DW)	
	M1	M2	M1	M2	M1	M2	M1	M2
0.000	7.00^d^	8.17^b^	17.84^d^	19.69^c^	43.06^e^	48.55^e^	33.29^c^	35.38^d^
0.025	14.10^a^	8.96^b^	21.07^a^	23.73^a^	50.45^b^	57.48^b^	37.07^a^	39.83^a^
0.050	12.00^b^	8.60^b^	20.24^ab^	23.14^ab^	52.19^a^	59.18^a^	36.27^a^	39.02^ab^
0.075	10.30^c^	9.15^b^	18.39^cd^	21.78^b^	46.94^c^	55.54^c^	34.18^bc^	37.66^c^
0.100	9.90^c^	10.80^a^	19.09^bc^	22.38^ab^	45.18^d^	53.57^d^	34.79^b^	38.32^bc^
LSD	0.86**	1.18**	1.20**	1.50**	1.22**	1.42**	0.93**	1.06**

Values marked with the different alphabetical letters within group of means refer to significant variations, using least significant difference (LSD) test; ** = Highly significant at 0.01 level of probability

#### Vegetative traits

All vegetative growth traits of marigold were significantly influenced by colchicine concentration in M1 and M2 generations, except stem diameter and leaves number plant^− 1^ in M1 generation and branches number plant^− 1^ in both generations (Table 4). In M1 generation, colchicine at a concentration of 0.075% showed the maximum increases of plant height, leaf area, fresh and dry weights of vegetative growth, statistically equaling the concentrations of 0.025% (for plant height, fresh and dry weights of vegetative growth) as well as 0.100% (for leaf area, and fresh and dry weights of vegetative growth). In M2 generation, all colchicine concentrations (0.025, 0.050, 0.075 and 0.100%) recorded similar increases in plant height, stem diameter and fresh weight of vegetative growth, surpassing the control treatment (0.00%). Furthermore, the concentration of 0.100% along 0.025% and 0.05% (for leaves number plant^− 1^), 0.00% and 0.075% (for leaf area) and 0.05% (for dry weight of vegetative growth) were the efficient treatments for recording the highest values.

**Table 4:** Mean values of vegetative growth traits of marigold as affected by colchicine concentration in the first (M1) and second (M2) generations

Colchicine %	Plant height (cm)		Stem diameter (cm)		Branches number plant^− 1^		Leaves number plant^− 1^		Leaf area (cm^2^)		Weight of vegetative growth (g)			
											Fresh		Dry	
	M1	M2	M1	M2	M1	M2	M1	M2	M1	M2	M1	M2	M1	M2
0.000	37.73^c^	36.00^b^	1.27^a^	1.12^b^	40.67^a^	40.33^a^	232.7^a^	249.0^b^	25.7^bc^	29.9^abc^	118.38^b^	87.15^b^	29.11^c^	24.00^c^
0.025	46.10^ab^	52.90^a^	1.28^a^	1.59^a^	45.67^a^	56.00^a^	280.1^a^	351.0^a^	16.6^c^	19.2^c^	172.67^a^	131.07^a^	49.03^a^	30.95^bc^
0.050	39.73^bc^	50.07^a^	1.13^a^	1.61^a^	48.57^a^	56.67^a^	280.8^a^	337.7^a^	25.8^bc^	23.2^bc^	121.39^b^	149.43^a^	33.77^bc^	51.77^a^
0.075	47.33^a^	45.40^ab^	1.45^a^	1.67^a^	48.89^a^	34.58^a^	270.4^a^	265.9 ^b^	30.9^ab^	30.9^ab^	159.73^a^	143.30^a^	46.87^ab^	35.90^bc^
0.100	39.97^bc^	52.73^a^	1.35^a^	1.65^a^	41.80^a^	61.80^a^	221.3^a^	328.5^a^	37.1^a^	41.8^a^	136.90^ab^	144.15^a^	37.43^abc^	43.20^ab^
LSD	6.98*	10.48*	NS	0.38*	NS	NS	NS	49.76**	10.74*	11.15*	37.81*	25.46**	13.91*	14.46*

Values marked with the different alphabetical letters within group of means refer to significant variations, using least significant difference (LSD) test; *= Significant at the 0.05 level of probability; *NS* Not significant

#### Flowering traits

As for flowering traits, colchicine treatments had a significant effect on all flowering attributes of marigold in M1 and M2 generations, except flowering date in M1 and inflorescence stalk length in both generations (Table 5). In M1 generation, application of 0.025% colchicine was the superior treatment for stimulating all inflorescence attributes with lowering flowering period. However, there were non-significant variations between 0.025% colchicine and each of 0.075% colchicine (for inflorescences number plant^− 1^), 0.100% colchicine (for fresh weight of 10-infloresence), and 0.075% or 0.100% colchicine (for inflorescence diameter). Moreover, the colchicine concentrations of 0.075% or 0.100% showed the longest flowering period in M1 generation of marigold. Concerning M2 generation, application of 0.100% colchicine achieved the highest values of flowering date and inflorescence attributes, while the longest flowering period was obtained with application of 0.025% colchicine. However, the differences between 0.100% colchicine and 0.000%, 0.050% or 0.075% colchicine (for flowering date), 0.025% or 0.075% colchicine (for inflorescence number plant^− 1^), 0.075% colchicine (for fresh weight of 10-infloresence), and 0.025%, 0.050% or 0.075% (for dry weight of 10-infloresence) were not significant.

**Table 5:** Mean values of flowering traits of marigold as affected by colchicine concentration in the first (M1) and second (M2) generations

Colchicine %	Flowering date (days)		Inflorescence attributes										Flowering period (day)	
			Number plant^− 1^		Weigh of 10-inflorescence (g)				Diameter (cm)		Stalk length (cm)			
					Fresh		Dry							
	M1	M2	M1	M2	M1	M2	M1	M2	M1	M2	M1	M2	M1	M2
0.000	23.67^a^	114.0^a^	57.2^b^	42.7^c^	11.6^bc^	9.9^c^	1.50^b^	1.29^b^	5.20^b^	5.63^bc^	4.56^a^	4.13^a^	176.0^c^	172.7^b^
0.025	21.67^a^	84.0^b^	80.8^a^	80.3^a^	14.2^a^	11.0^bc^	1.76^a^	1.54^a^	5.49^ab^	5.64^bc^	4.69^a^	3.78^a^	180.0^bc^	211.0^a^
0.050	25.33^a^	107.0^a^	63.3^b^	55.0^bc^	10.4^c^	10.0^c^	1.37^b^	1.50^ab^	5.24^b^	5.85^b^	4.20^a^	3.98^a^	177.3^bc^	182.7^b^
0.075	23.00^a^	107.0^a^	78.8^a^	62.2^abc^	12.4^b^	12.2^a^	1.48^b^	1.64^a^	5.75^a^	5.51^c^	4.83^a^	4.29^a^	182.3^ab^	178.0^b^
0.100	19.33^a^	114.7^a^	60.1^b^	76.1^ab^	12.9^ab^	11.8^ab^	1.15^c^	1.65^a^	5.57^a^	6.12^a^	4.61^a^	3.80^a^	186.0^a^	180.7^b^
LSD	NS	7.9**	15.1*	21.7*	1.6**	1.2**	0.22**	0.22*	0.32*	0.26**	NS	NS	5.0*	17.9**

Values marked with the different alphabetical letters within group of means refer to significant variations, using least significant difference (LSD) test; * and **= Significant at the 0.05 and 0.01 level of probability; *NS* Not significant

#### Abnormalities

**Fig. 3:**
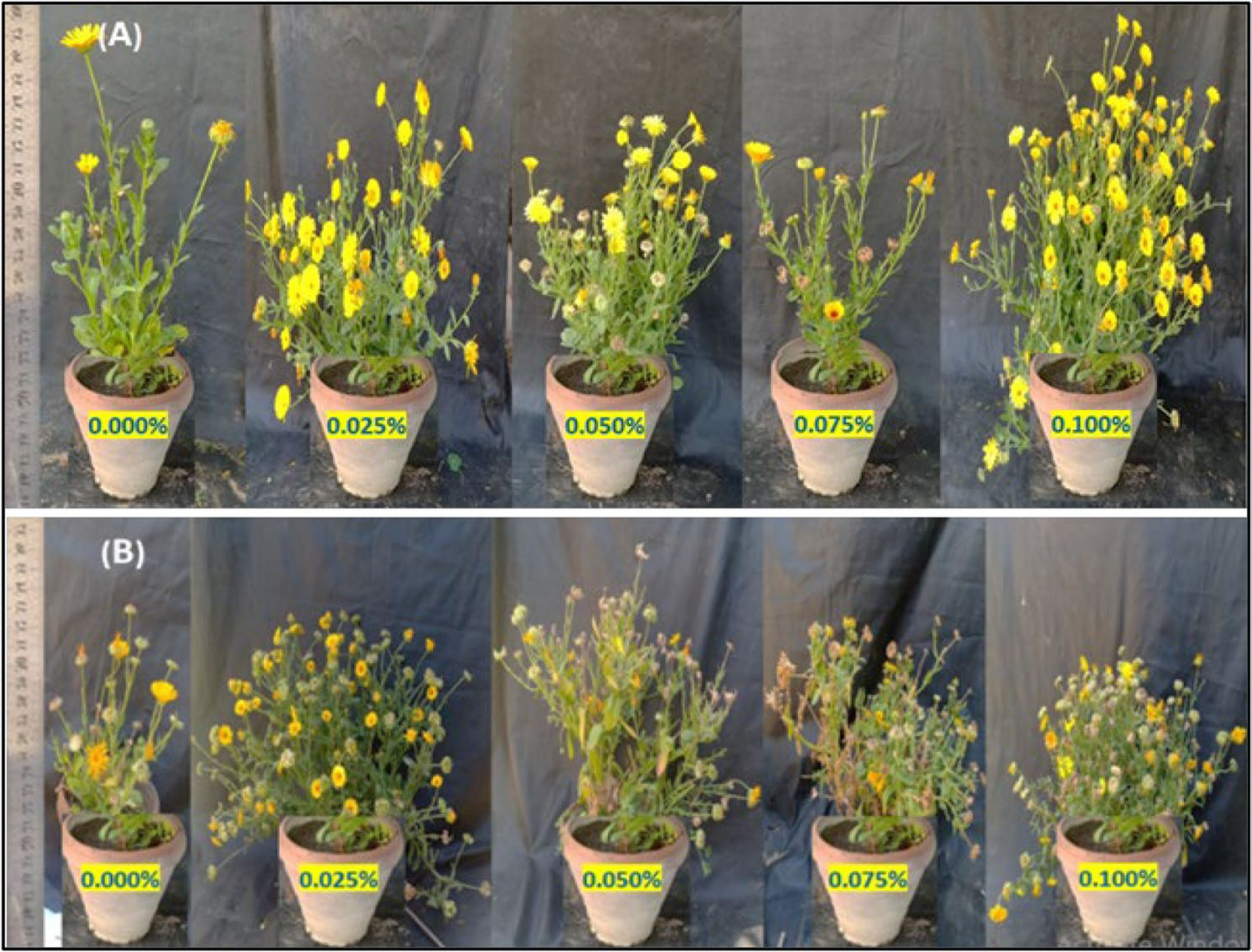
A Photograph showing change in the growth habit (branching) in the first (**A**) and second (**B**) generations of marigold plants as a result of the treatment with colchicine at 0.000, 0.025, 0.050, 0.075 and 0.100%

**Fig. 4:**
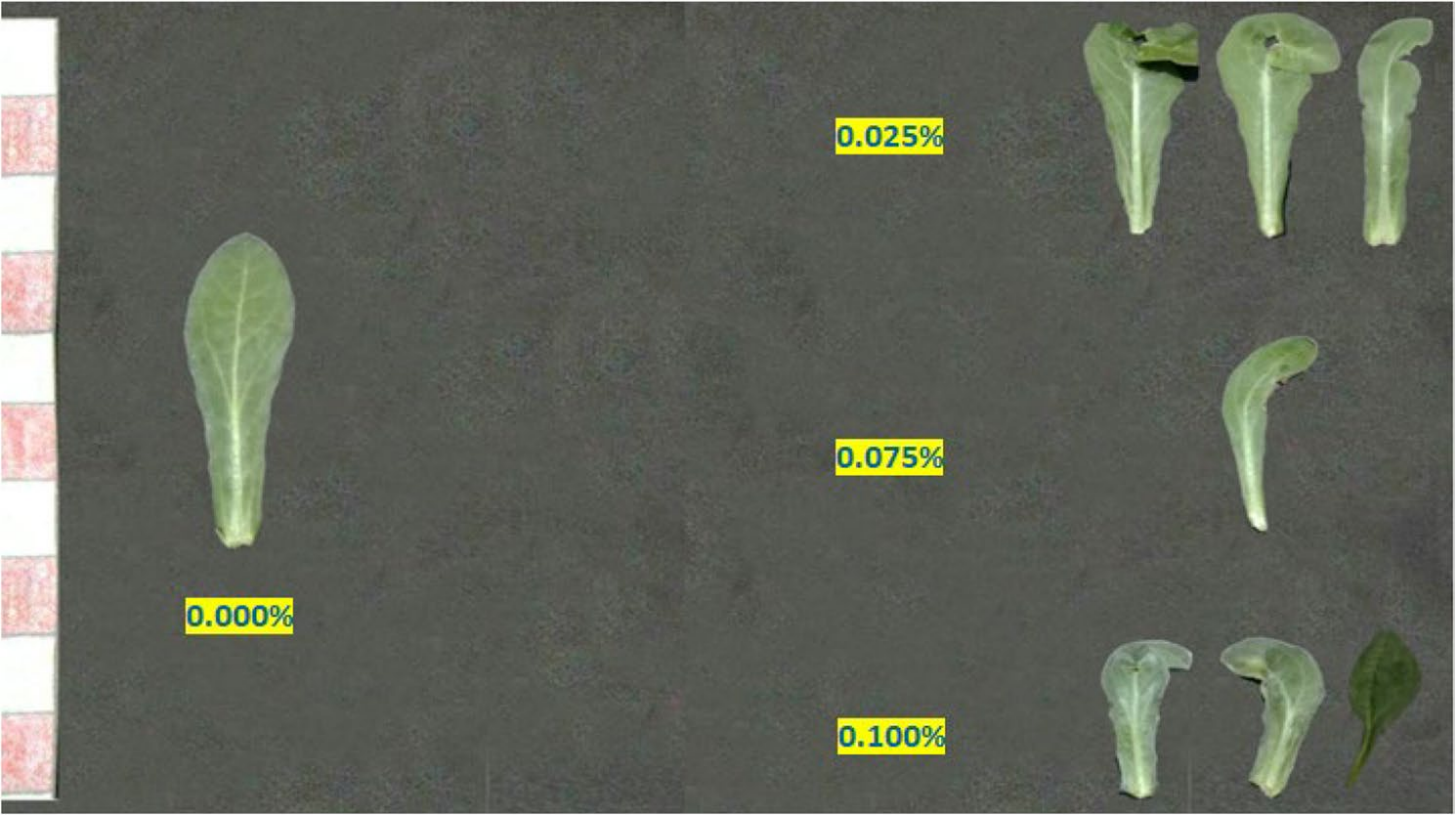
A photograph showing leaf shapes abnormalities in the first generation of marigold plants as a result of the treatment with colchicine at 0.000, 0.025, 0.075 and 0.100%

**Fig. 5:**
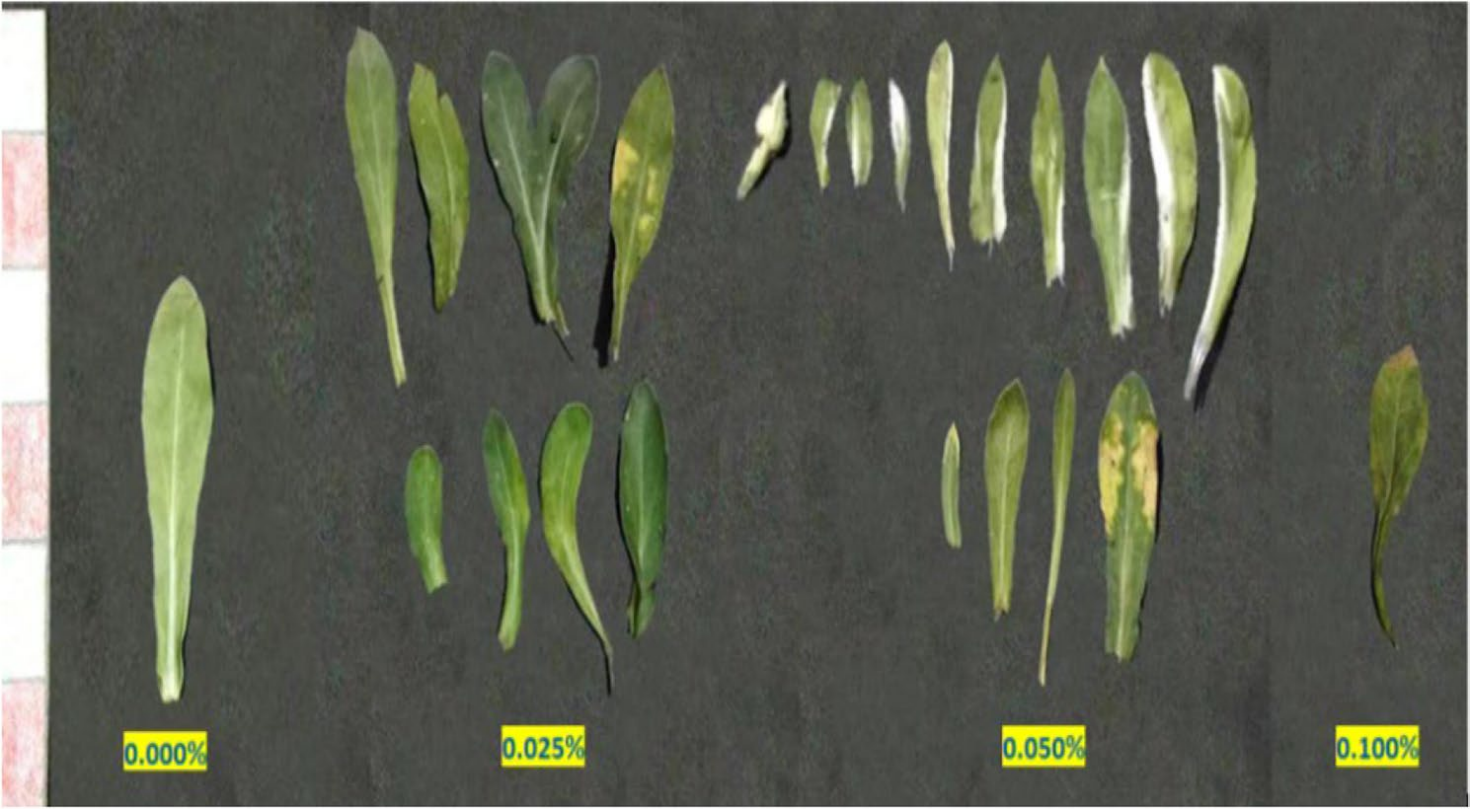
A photograph showing the leaf color abnormalities in the second generation of marigold plants as a result of the treatment with colchicine at 0.000, 0.025, 0.050 and 0.100%

**Fig. 6:**
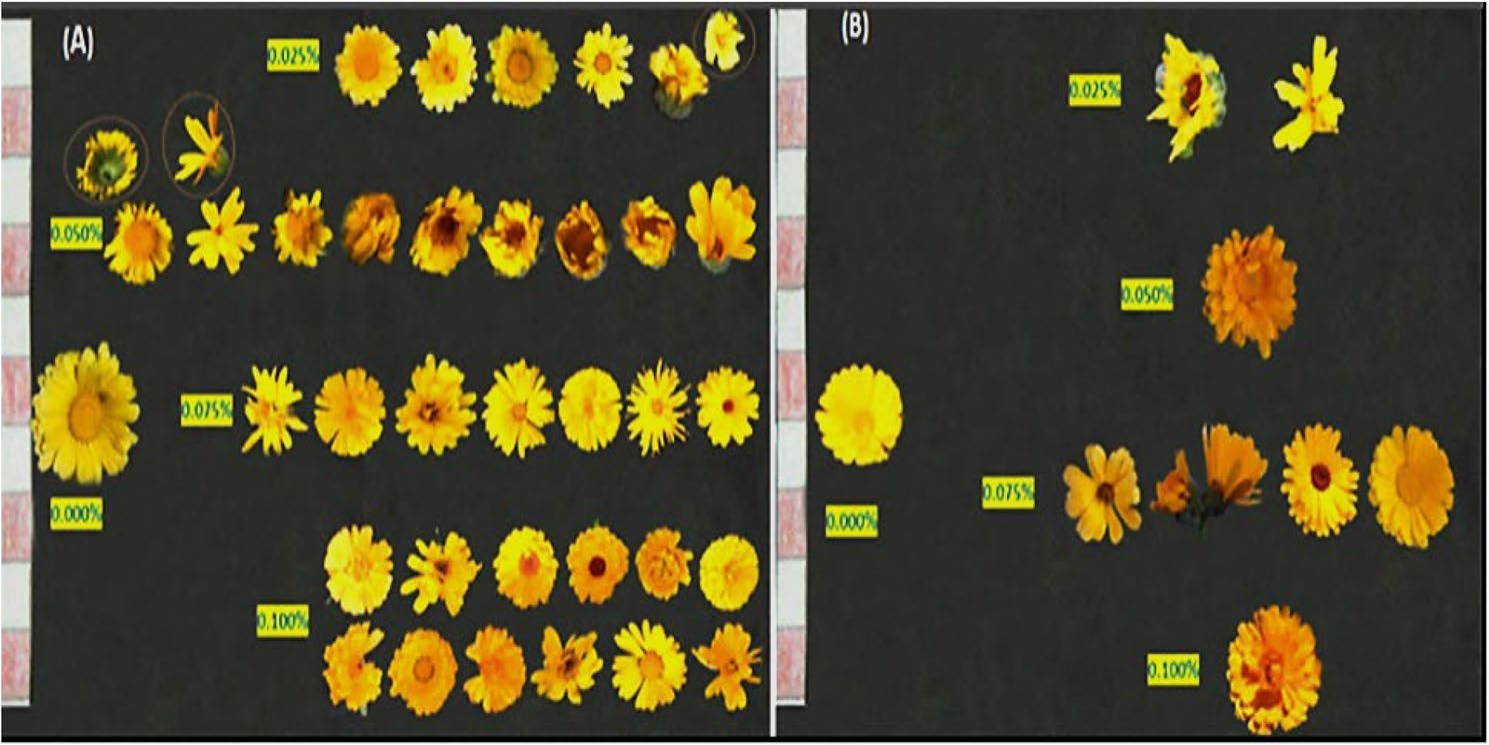
A photograph showing changes in the inflorescence forms in the first (**A**) and second (**B**) generations of marigold plants as a result of the treatment with colchicine at 0.000, 0.025, 0.050, 0.075 and 0.100%

The variations induction (aberrations) of colchicine expressed in changes in growth habit, leaf abnormalities, and inflorescences abnormalities were monitored. Colchicine treatment induces significant alterations in plant growth architecture, including dwarfism, bushiness, or altered branching patterns (Fig. 3; Table 6). Colchicine application at 0.025% and 0.075% showed dwarfed plants joined with reddish leave on the former treatment. In M1 generation plants, dwarfism primarily arises from physiological damage (e.g., microtubule disruption impairing cell division), which is often transient. Both 0.050% and 0.100% concentrations showed abnormal and multiplied branching. Leaf deformities (e.g., altered lamina symmetry, thickened blades, or serration defects) in M2 plants stem from chromosomal aberrations (aneuploidy, translocations) and sectorial chimerism in meristematic layers (Figs. 4 and 5). Inflorescence aberrations (e.g., fascinated stems, floral gigantism, or reduced floret numbers) result from colchicine’s dose-dependent disruption of meristem organization (Fig. 6).

**Table 6:** A descriptive analysis of mutations resulting from different concentrations of colchicine in marigold plants

Colchicine %	Change
0.000%	Normal
0.025%	Dwarfed plant
0.050%	Strange branching -Taller plant
0.075%	Dwarfed plant and leave color of red
0.100%	Strange branching

### Polymerase chain reaction (PCR) analysis

**Fig. 7:**
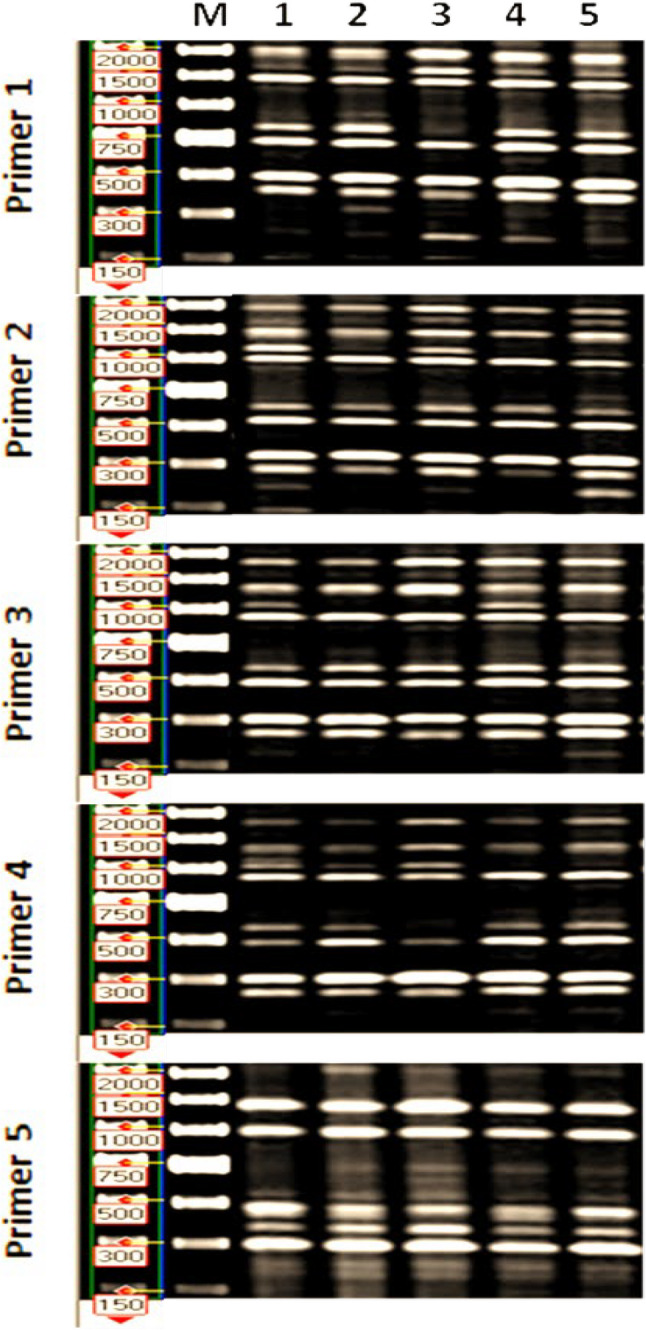
PCR amplified fragments for the control (original parent) and variant plants of marigold plants after amplification with the primer ISSR (1–5)

Inter simple sequence repeat (ISSR) was applied to evaluate genetic diversity among five mutants of marigold plants obtained from colchicine treatments (Fig. 7; Supplementary Fig. 1). Five primers were used and generated 65 amplified bands, 26 of them were polymeric with 39.7% polymorphism percentage as shown in Table 7. The fourth primer generated the highest polymorphism (53.8%) compared to 50, 35, 35, 25% for first, second, third and fifth primer respectively. The control plant showed the highest genetic similarity with the mutant 4 (94.7) followed by mutants 3 and 5 (90), while the lowest value was obtained with mutants 2 (87.7) as shown in Table 8.

**Table 7:** Number of amplified bands, number of polymorphic band and primer polymorphism % detected by ISSR marker in the M2 generation of marigold plants .as affected by colchicine treatments

Primer no.	Number of amplified band	Number of polymorphic band	Primer polymorphism%
1	2	6	50.0
2	14	5	35.0
3	14	5	35.0
4	13	7	53.8
5	12	3	25.0

**Table 8:** Genetic distance of DNA among M2 generation of marigold plants treated by colchicine using ISSR marker

Primer no.	1	2	3	4	5
1	100.0				
2	87.7	100.0			
3	90.0	87.9	100.0		
4	94.7	86.8	82.3	100.0	
5	90.0	87.6	81.4	88.8	100.0

**Fig. 8:**
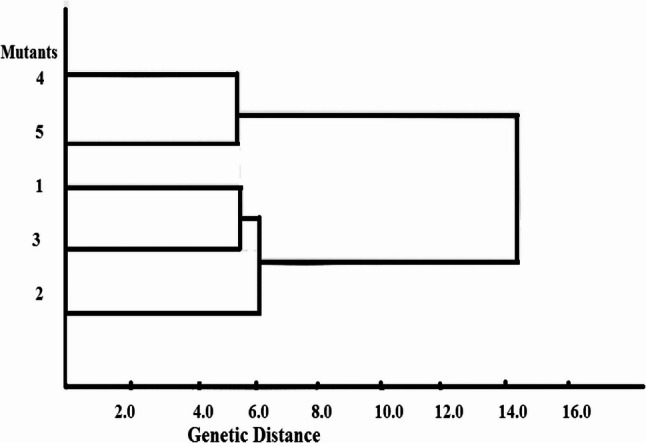
Tree diagram for the second generation of marigold plants treated by colchicine on the basis of ISSR using profile five ISSR marker

Phylogenetic tree identified two groups (Fig. 8) the mutant. The mutant 2 was more distinct genetically from mutant 1 and 3. These abovementioned result refer to the effect of colchicine inducing mutants in marigold plants in which an ISSR technique able to distinguish among them.

## Discussion

### Survival and germination percentages

Results of survival % are consistent with the findings of Jeloudar et al. [43]. Shoot-tip application specificity, localized treatment, minimizes systemic toxicity compared to whole-plant exposure [44]. Based on the critical threshold effect, survival may depend more on exposure duration than concentration, as colchicine’s efficacy plateaus beyond certain thresholds [45]. According to genotype-dependent resilience, plant species often exhibit inherent differential tolerance to polyploidizing agents [46], potentially buffering concentration effects. Higher concentrations often reduce seedling viability due to mitotic disruption [47].

The reduction in germination rates in the present study can be attributed to colchicine’s known impact on meristematic tissues, where it disrupts normal mitotic division. As Raina et al. [48] reported, high doses of mutagens can lead to cellular death, physiological dysfunctions, and chromosomal damage during seed germination. Chromosomal aberrations in the meristematic tissues can severely impact enzymatic activities essential for germination, including those of catalase and lipase [49]. Furthermore, Singh et al. [50] highlighted that such genetic disturbances can also trigger hormonal imbalances. These hormonal changes, such as reduced auxin synthesis or disrupted hormonal signaling, are critical during the early stages of seedling development, as supported by the findings of Lewis et al. [51]. Roychowdhury and Tah [52] suggested that reduced germination and early plant vigor under mutagenic stress may result from sudden shifts in metabolic activity, inhibition of auxin production, degradation of growth inhibitors, or changes in the balance of growth-promoting substances. A decline in the seed’s assimilation capacity under colchicine influence may also contribute to poor germination performance. The data suggest that colchicine exerts a significant ISSR-detected genetic polymorphism on seed germination in marigold through interference with cell division, enzymatic activity, and hormonal regulation. The sensitivity of seed germination to colchicine treatment emphasizes the need for optimizing mutagen doses to balance mutation induction and seed viability in future breeding programs.

### Plant pigments

Colchicine can alter chlorophyll pigment levels, with its effect varying depending on concentration and generation. The higher concentrations, such as 0.075% in M1 and 0.100% in M2, tended to enhance chlorophyll a levels, while certain concentrations (e.g., 0.050%) could reduce chlorophyll b. Such variations may be linked to colchicine-induced polyploidy, which can modify chloroplast size, number, and metabolism, thereby influencing pigment biosynthesis. Increased chlorophyll content is often associated with improved light-harvesting capacity and higher photosynthetic efficiency, which can contribute to greater biomass production in polyploid plants. These observations are in agreement with those reported by El-Nashar and Ammar [18] and Fahmy et al. [53] in marigold who found that colchicine treatments could enhance chlorophyll content, particularly at moderate doses. Recent research corroborates these findings; for instance, Malabadi et al. [54] reported that colchicine-induced polyploidy in *Cannabis sativa* led to elevated chlorophyll concentrations. This increase was attributed to the presence of larger chloroplasts and the up-regulation of chlorophyll biosynthetic enzyme activity. Tetraploid of *Stevia rebaudiana* plants had a significantly higher chlorophyll content indicating higher photosynthetic capacity than the diploid controls [55].

The results of carotenoid partially align with those reported by Fahmy et al. [53] for marigold where colchicine treatments at optimal concentrations enhanced carotenoid accumulation, likely due to increased plastid size and altered metabolic activity in polyploid cells. Similarly, Robu et al. [56] found an increase in carotenoid content when colchicine was applied at 400 ppm, suggesting that the effect is concentration-dependent. In contrast, El-Nashar and Ammar [18] observed no significant differences in total carotenoid content between colchicine-treated plants and the control, indicating that species-specific responses and environmental factors may influence pigment accumulation. From a physiological perspective, colchicine-induced polyploidy can alter pigment biosynthesis pathways through gene dosage effects and changes in chloroplast structure. Moderate doses may stimulate carotenoid production by increasing the expression of genes in the terpenoid biosynthetic pathway, while excessively high or prolonged exposure could cause metabolic imbalances, oxidative stress, or impaired pigment synthesis [57]. The absence of significant differences in the M2 generation could be attributed to genetic stabilization over generations, where the initial mutagenic stimulus diminishes as the plant population segregates. Physiologically, tetraploids demonstrated increased stomatal size and chloroplast count in stomata but reduced stomatal density. Nutrient analysis revealed a substantial increase in polysaccharides, calcium, iron, and zinc in tetraploid leaves. In addition, seventeen carotenoids were identified in the leaves of *Lycium chinense*. Recent studies also confirm that the effect of colchicine on secondary metabolites, including carotenoids, is highly variable. For example, Zhang et al. [58] reported that tetraploids exhibited higher levels of carotenoid accumulation compared to the diploid. This underscores the need for optimization of treatment concentration and exposure time to maintain pigment quality and yield. Carotenoid content in *Catharanthus roseus* (L.) G. Don tetraploid leaves increased by 1.42 and 1.34 times, respectively [59]. The upregulation of chlorophyll and carotenoid biosynthesis genes, combined with the downregulation of their degradation genes following genome duplication, likely accounts for the observed increase in these essential pigments [60].

### Total soluble carbohydrates and phenols

The results of carbohydrates are consistent with previous findings in marigold [61], *Echinacea purpurea* [62], and *Salvia leriifolia* [63], where colchicine-induced polyploidy modulated carbohydrate metabolism. The enhancement of carbohydrate content at optimal concentrations may be attributed to increased leaf area and improved photosynthetic capacity due to enlarged cell size and altered gene expression in polyploids. However, higher colchicine concentrations can cause metabolic stress, disrupt enzymatic activity in carbohydrate synthesis pathways, and lead to reduced carbohydrate accumulation. These findings supported by Hassan et al. [61]. that demonstrated that the moderate colchicine treatments in marigold enhanced carbohydrate levels due to improved photosynthetic pigment content and sink–source balance, while higher doses impaired carbohydrate biosynthesis.

Results of total phenols in the marigold leaves align with those of El-Nashar and Ammar [18] Fahmy et al. [53] on marigold where colchicine application influenced phenolic compound biosynthesis. The increase in phenolic content at higher colchicine concentrations may be associated with the activation of secondary metabolite pathways as part of the plant’s stress response to chromosomal doubling. This is supported by the fact that polyploid plants often exhibit higher concentrations of bioactive compounds due to increased gene dosage and altered metabolic regulation. Gupta et al. [64] reported that colchicine-induced polyploidy in *Nigella sativa* significantly increased phenolic content and antioxidant activity, attributing the enhancement to upregulation of the phenylpropanoid pathway under polyploid conditions. The elevated antioxidant capacity of the plant may be due to the caused stress by mutation owing to colchicine treatment. Our results are in virtuous agreement with previous findings where the levels of flavonoid, phenolics, and total antioxidant capacity were affected by colchicine treatment [65].

#### Biochemical constituents in inflorescence

It has been noted that increasing colchicine concentration generally enhanced β-carotene accumulation in *Echinacea purpurea* [62] and *Sesamum indicum* [66]. Overall, the results support the hypothesis that colchicine-induced polyploidy can be an effective strategy to enhance β-carotene and total carotenoid content in ornamental species, potentially improving their nutritional, medicinal, and aesthetic value. However, the response is dose-dependent and may differ between generations, highlighting the importance of optimizing treatment concentration and evaluating long-term stability of the trait. The increase in β-carotene content observed in this study can be attributed to colchicine’s mode of action as a mitotic inhibitor that disrupts spindle fiber formation, leading to chromosome doubling and polyploidy induction [46]. Polyploid plants often exhibit larger cells, increased metabolic capacity, and altered biosynthetic pathways, which can enhance the synthesis of secondary metabolites such as carotenoids [54]. β-Carotene, being a precursor of vitamin A and a major pigment contributing to flower coloration, may accumulate in greater amounts in colchicine-induced polyploids due to increases the number of gene copies encoding enzymes in the carotenoid biosynthetic pathway, potentially boosting production rates [58] as well as increases plastid size and number, which are the primary sites of carotenoid storage [54]. Also, changes in plant growth dynamics via altered metabolic allocation may channel more carbon skeletons toward pigment biosynthesis [56]. Enhanced secondary metabolite accumulation in colchicine-treated plants was observed [53]. Also, Robu et al. [56], documented increases in flavonoid and phenolic contents following 400 ppm colchicine application.

Colchicine is a well-known antimitotic agent that disrupts spindle fiber formation during cell division, leading to chromosome doubling and the induction of polyploidy [18]. Polyploidization often enhances metabolic activity, increases cell size, and alters the biosynthetic capacity of plants. This can result in higher accumulation of secondary metabolites, including coumarins, terpenoids, flavonoids, and carotenoids. The observed increases in coumarin, terpenoid, and flavonoid contents under colchicine treatments suggest that genome duplication may have stimulated key biosynthetic pathways, possibly by increasing gene dosage and enzymatic activity involved in the phenylpropanoid and terpenoid pathways. Enhanced pigment synthesis, including carotenoids, is also a common outcome of polyploidy due to both structural (larger chromoplasts) and metabolic (upregulated isoprenoid pathway) changes [58]. Therefore, it is reasonable to expect that colchicine treatment would also have a notable impact on total carotenoid content in marigold. Carotenoids, as terpenoid derivatives, share biosynthetic precursors with other isoprenoids; thus, the observed rise in total terpenoids at 0.050% colchicine may indicate parallel stimulation of carotenoid biosynthesis. This could translate into enhanced flower coloration, improved antioxidant capacity, and potentially higher medicinal value of the inflorescences.

#### Vegetative traits

The results of vegetative growth were similar to those reported on *Nigella sativa* L [28]. and on *Calendula officinalis* L [18]. in which, artificial induction of mutations by colchicine leads to an alteration of the plant genome through an increased cellular division rate and an expansion of the meristematic regions, probably through alterations of the signaling pathway [67]. Nura et al. [66] reported significant increases in plant height of colchicine treated sesame plants. In contrast, the findings of Maluszynski et al. [68] showed a noticeable decrease in the height of rice plant, as a result of induced mutation. This discrepancy is resolved by Heslop-Harrison et al. [69], stating that pan-species analysis revealed that dicots with indeterminate growth (like *Calendula* and *Sesame*) exhibit ploidy-correlated stem elongation through cytokinin hypersensitivity: Colchicine upregulates *ARR* response regulators [70]; and vascular expansion: Polyploidy induces wider xylem vessels [71]. The colchicine may have influenced cytokinin activity, which is essential for plant development [72]. The present study showed that colchicine treatments resulted in an increase in both the number of leaves per plant and in leaf area. This is in accordance with the findings of Nura et al. [73], who found an increase in leaf number and area in jute plant mutants. An increase in leaf number provides an increase in the surface for gaseous exchange that has a considerable effect on the photosynthesis [74]. Also, data results exhibited the beneficial influence of colchicine on marigold biomass. These findings align with El-Nashar and Ammar [18] on marigold where optimized colchicine levels boosted biomass. The generational divergence likely stems from colchicine’s cumulative ISSR-detected genetic polymorphism. As a polyploidy-inducing agent, prolonged colchicine exposure alters morphological traits across generations [45]. Mid-range treatments (e.g., 0.025–0.050%) may optimize polyploidy-induced biomass gains by balancing mutagenic efficiency and phytotoxicity [57]. Critical factors—including exposure time, pH, temperature, and seed pretreatment—further modulate these responses [75, 76], explaining the treatment-specific efficacy observed here.

### Flowering traits

The absence of M1 effects on flowering date suggests immediate physiological responses may mask genomic changes, while M2 alterations confirm stable genetic modifications, ISSR-detected genetic polymorphism, induced by colchicine [77]. This generational divergence aligns with Maamoun [28] observations in *Nigella sativa* and reflects fundamental principles of mutagenesis. The flowering delay at 0.100% colchicine concentration exemplifies the trade-off between mutagenic enhancement and physiological stress: higher concentrations disrupt developmental timing by interfering with floral integrator genes (e.g., *FT*, *SOC1*) while altering hormone signaling pathways [78]. Recent studies confirm that optimal colchicine doses (typically 0.01–0.05%) accelerate flowering in polyploids, but supra-optimal concentrations (> 0.100%) delay reproductive transition through epigenetic modifications of flowering-time genes [76, 79]. This generational divergence in optimal concentration mirrors dose-dependent flowering time alterations in polyploid *Cannabis*, where low-level mutagenesis prolonged reproductive phases via flowering locus *C* modulation [80].

In the M1 generation, 0.025% colchicine treatment significantly increased inflorescence number compared to the, with 0.075% yielding intermediate results. Other concentrations showed no statistical difference from controls. Similarly, in M2, 0.025% colchicine followed by 0.100%, producing the maximal inflorescences. These results align with dose-dependent colchicine effects reported in marigold where low concentrations enhanced flower induction, while higher doses suppressed reproductive development in both M1 and M2 generations [18] and recent *Chrysanthemum* studies where polyploidization enhanced floral organ size through cell expansion mechanisms [81]. This biphasic response mirrors findings in *Vigna mungo* [82] Mitotic chromosome through colchicine treatment results in production of large sized inflorescence with increased floral parts in salvia (*Salvia coccinea* cv. Coral Nymph), however flowering has been delayed up to 10–30 days [83]. Lower colchicine concentration led to an increase in the flowers number plant^− 1^. However, elevated colchicine levels led to a reduction in the number of flowers in the M1 and M2- generation, which agrees with the findings on *Vigna mungo* [82]. The emergence of late and early flowering Calendula mutants in the M1 and M2 -generation found in our study was similar to that of [24, 52, 84]. The strong ISSR-detected genetic polymorphism on this trait is probably due to the tendency of the mutagen to alter gene(s) responsible for inducing flowering, by altering plant response to environmental signals [51].

The presented data indicated significant variation in the fresh weight of 10-inflorescence across colchicine treatments and generations. In the M1 generation, the 0.025% colchicine treatment yielded the highest fresh weight, significantly exceeding the control. Conversely, the 0.050% treatment produced the lowest fresh weight. In the M2 generation, the 0.075% colchicine treatment resulted in the highest fresh weight, surpassing the control, followed by the 0.100% treatment. Dry weight responses mirrored fresh weight trends but exhibited distinct optima. In M1, the 0.025% colchicine treatment generated the highest dry weight significantly greater than control and all other concentrations. In M2, the maximum dry weight was observed under the 0.100% colchicine treatment, significantly higher than the control. This differential response between generations aligns with established patterns of mutagen-induced phenotypic variation in plants, where optimal concentrations often shift due to altered genomic stability [85]. This generational divergence in optimal concentrations aligns with findings on colchicine-induced ploidy stability in *Brassica* spp., where M2 phenotypes often exhibit altered dose responses due to meiotic irregularities [86]. This concentration-specific generational shift reflects tetraploidy-associated resource allocation trade-offs, where polyploid M2 plants prioritize structural biomass over water retention—a phenomenon documented in *Solanum tuberosum* polyploids [87]. The significant dry weight increasing (*p* < 0.05) at optimal doses underscore colchicine’s role in enhancing assimilate partitioning to reproductive structures, consistent with cytochimera-driven sink strength modulation [88].

In the M1 generation, the widest inflorescence diameters were recorded at 0.075% and 0.100% treatments, significantly exceeding the control. The M2 generation exhibited further enhancement, with the 0.100% treatment yielding the largest diameter. These results align with colchicine-induced polyploidy studies reporting gigantism in floral organs, such as those in marigold [18]. Also, He et al. [89] on *Tagetes erecta* tetraploids corroborate this trend, attributing increased inflorescence size to cell expansion and endoreduplication.

No significant differences occurred in inflorescence stalk among treatments in either generation, indicating trait stability under polyploidization. This parallels findings in *Dendranthema grandiflora* [90] and controversy tetraploid was shorter than that of diploid control plants in *Dendranthema indicum* var. *aromaticum* [91], where architectural traits like stalk length were affected by genomic duplication.

The M1 generation showed maximal flowering duration under 0.100% treatment vs. control. In M2, the 0.025% treatment significantly extended flowering to 211.00 days (control: 172.67 days). This aligns with polyploidy-induced delays in senescence, as reported in *Echinacea purpurea* tetraploids by Abdoli et al. [62]., where genome duplication altered ethylene-responsive genes and prolonged reproductive phases.

As for the abnormal growth, heritable changes persisting into M2 generations indicate genomic mutations affecting genes regulating growth hormones (e.g., gibberellin biosynthesis) or meristematic activity [92]. Recent genomic studies confirm that colchicine-induced polyploidy alters expression patterns of growth-related genes (e.g., GA20-oxidase), leading to phenotypic variants [62, 93]. Colchicine disrupts mitotic spindle assembly, causing irregular chromosome segregation and somatic mutations. This genomic instability reshapes leaf developmental pathways, notably affecting auxin-mediated venation patterning and KNOX gene expression [94, 95]. Advanced cytogenomic analyses reveal that polyploidy-induced epigenetic modifications, further suppress leaf-size regulators [96, 97]. Low concentrations (0.01–0.1%) perturb cell cycle dynamics (increasing mitotic index), while high concentrations (0.5–1.0%) cause endopolyploidy, altering floral organ size via unbalanced cell expansion/proliferation [53, 98]. The changes to dysregulation of floral identity genes (e.g., *LFY*, *AP1*) and auxin efflux carriers (PIN proteins), leading to abnormal phyllotaxis [99]. A delay in flowering, with increase in flower diameter and malformation of flowers along with floral parts of different colors on the same branch, are the common characteristics of colchicine treated plants [100].

The difference in optimal colchicine concentration between the M1 generation (0.025%) and M2 generation (0.100%) may be attributed to the complex biological nature of induced mutagenesis. The favorable response at lower concentrations in M1 likely reflects transient somatic alterations or ‘physiological shock’ following treatment. However, the shift in M2 suggests the possible segregation of induced mutations and the gradual fixation of stable polyploid states, or conversely, the instability of certain chromosomal rearrangements that do not persist beyond the first generation. These generational contrasts highlight that phenotypic variation in the early stages of mutation breeding may not always reflect stable genetic gains, requiring further assessment to distinguish between transient somatic effects and fixed, heritable polyploidy.

### Inter simple sequence repeat (ISSR)

The utilization of molecular markers has become a cornerstone in assessing genetic diversity and evaluating the impact of mutagens in plant breeding programs. In this study, the ISSR technique was employed due to its cost-effectiveness, high reproducibility, and ability to provide informative displays of differences between closely related individuals. This is a low-cost method that uses a minimal amount of template DNA and was created by Zietkiewicz et al. [101] to find polymorphisms in microsatellites. Large numbers of fragments and reproducibility are produced using ISSR [102]. Primers used in this method typically amplify the sequence between two microsatellites and are based on a repeating sequence range by degenerate 3′ anchor [103]. In order to create new markers, gene focused markers are currently highly popular. These markers produce polymorphisms from the exon, intron, or promoter regions of the genes that may be directly related to the functioning of the genes [104]. This information is consistent with the previous findings of Ali et al. [105]. and Yilmaz and Ciftci [106].

### Colchicine-induced smorphological variation

The results confirm that colchicine acts as a potent mutagen in marigold (*Calendula officinalis* L.), inducing a wide range of morphological changes in the M2 generation. The observed abnormalities that including dwarfism, strange branching patterns, and alterations in leaf pigmentation—suggest that colchicine concentrations significantly influence marigold physiology and growth behavior. Specifically, the emergence of a taller plant with strange branching at 0.050% concentration versus dwarfed plants at 0.025% and 0.075% indicates a non-linear, dose-dependent response to the mutagen. These findings align with the hypothesis that colchicine can be used to create novel phenotypic variations in vegetative and flowering components.

### Genetic diversity and ISSR efficiency

The genetic analysis provided deep insight into the molecular shifts underlying these physical changes. The five ISSR primers generated a total of 65 bands, with an average polymorphism of 39.6%. This level of polymorphism highlights the effectiveness of ISSR markers in identifying genetic variations induced by chemical mutagens in marigolds.

### Primer performance

Primer 4 was identified as the most informative, yielding the highest polymorphism rate of 53.8%, which suggests it is particularly sensitive to the genomic regions altered by colchicine in this species.

### Genetic relationships

The similarity matrix revealed that while some mutants remained closely related to the control (e.g., Mutant 4 at 94.7% similarity), others showed significant divergence. Mutant 2 stood out as the most genetically distinct individual (87.7% similarity to control), corroborating its position in the phylogenetic tree.

### Correlation between morphology and genotype

The clustering of the mutants into two distinct groups in the phylogenetic tree reflects the underlying genetic distance created by the treatment. The ability of the ISSR technique to distinguish between these mutants proves that the morphological variations observed (such as the dwarfed stature of Mutant 2) are linked to detectable genomic changes rather than transient physiological stress. This is consistent with previous research utilizing gene-focused markers to identify polymorphisms in exons or introns that directly relate to plant functioning [108–110].

### Study limitations

While the present study provides valuable insights into the induction of genetic variability using colchicine, certain limitations should be acknowledged. Firstly, the assessment of polyploidy was based on morphological and molecular markers without direct cytological confirmation (e.g., chromosome counting or flow cytometry), which remains the definitive method for verifying ploidy levels. Secondly, the evaluation was restricted to the M1 and M2 generations; therefore, the long-term genetic stability and the commercial viability of the identified mutants require further investigation over subsequent generations. Future research incorporating cytogenetic analysis and multi-location field trials will be essential to validate these findings and ensure the stability of the improved traits.

## Conclusions

The obtained results indicated that different concentrations of colchicine caused some morphological variations in the vegetative, flowering growth as well as increased chemical composition of marigold plants. However, application of 0.025% colchicine for the first and second generations of marigold resulted in lowering phenolic content while increased total carbohydrates, thereby stimulating all inflorescence attributes with lowering flowering period. ISSR-PCR technique was able to detect mutations induced by colchicine application in marigold plants.

## Supplementary Information

Supplementary Material 1.

## Data Availability

The datasets used and/or analyzed during the current research are available from the corresponding author upon reasonable request.
