# Supplementary material for: Mutagenic and physiological impacts of colchicine on growth, flowering behavior, and ISSR-based genetic variability in Calendula officinalis L
Source: BMC Plant Biol. 2026 Apr 1;26:643. doi: 10.1186/s12870-026-08536-4 (PMC13063666; doi:10.1186/s12870-026-08536-4)
Supplement: Supplementary file 1 — Supplementary Material 1. [file 12870_2026_8536_MOESM1_ESM.docx]

**Primer 1 Primer 2 Primer 3 Primer 4 Primer 5**


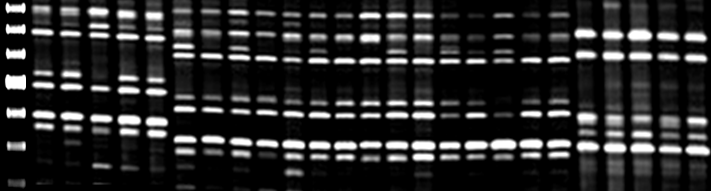

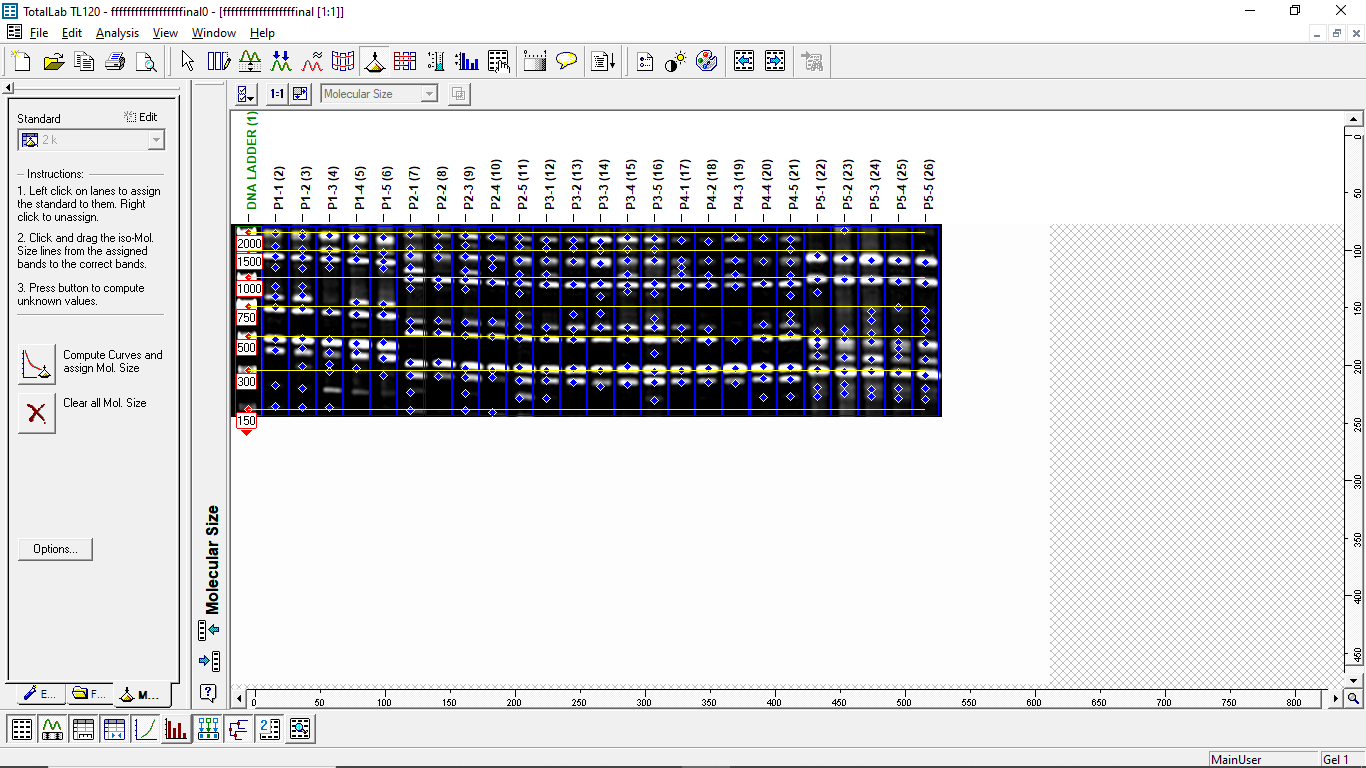


Supplementary Fig 1: Original images of PCR amplified fragments for the control (original parent) and variant plants of marigold plants after amplification with the primer ISSR (1-5).
